# Supplementary material for: Risk factors for sacrococcygeal pilonidal sinus: a systematic review and meta-analysis supplemented by genetic causal assessment
Source: Front Surg. 2026 Jan 7;12:1718589. doi: 10.3389/fsurg.2025.1718589 (PMC12819706; doi:10.3389/fsurg.2025.1718589)
Supplement: Supplementary file 2 [file Datasheet2.zip › Supplementary Data 2/MR_pipeline_p5e-6/leaveone_plots/cngb-met112-90.finngen_R12_L12_PILONIDALCYST.pdf]

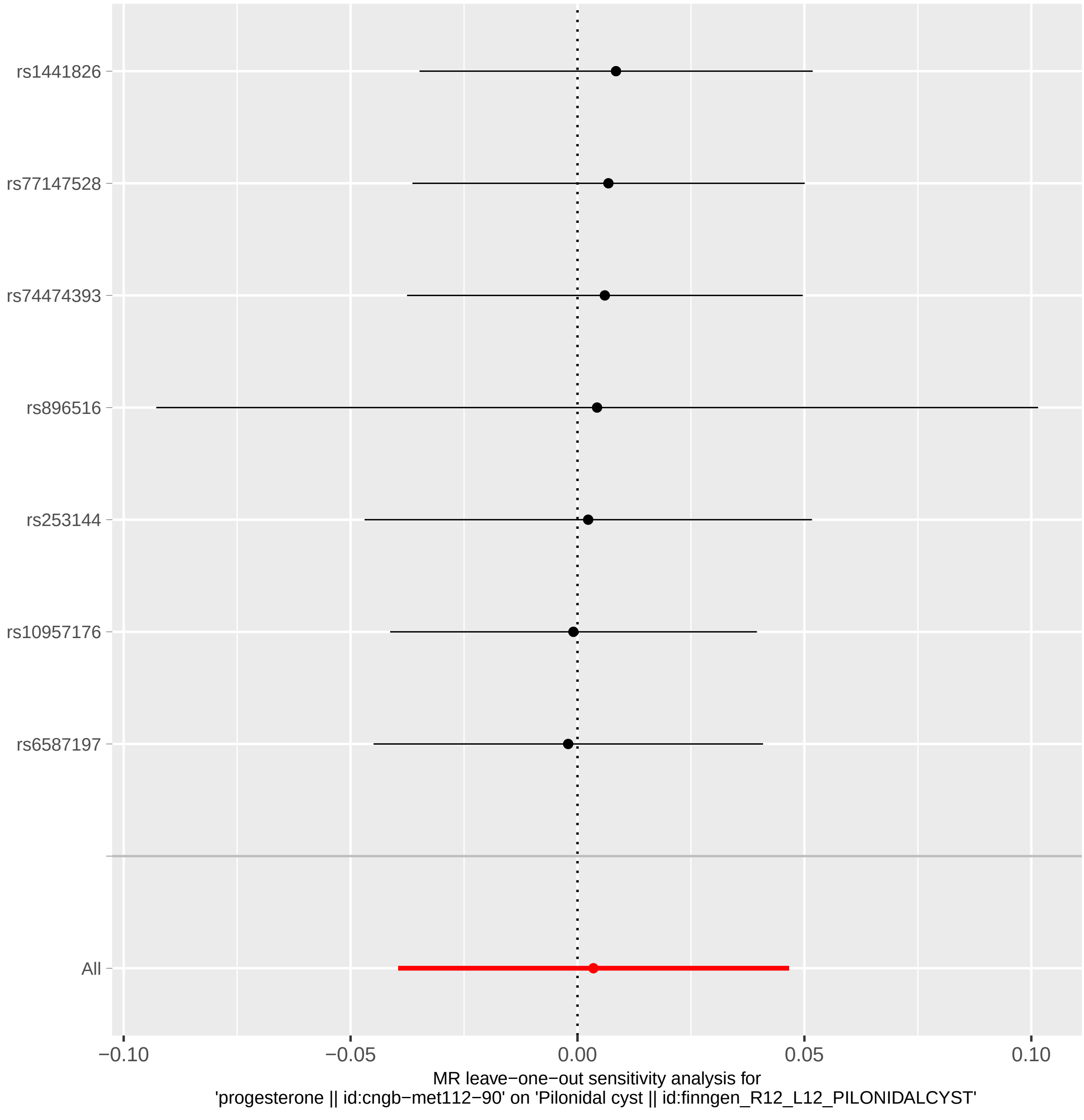
rs1441826
rs77147528
rs74474393
rs896516
rs253144
rs10957176
rs6587197
All
−0.10
−0.05
0.00
0.05
0.10
MR leave−one−out sensitivity analysis for
'progesterone || id:cngb−met112−90' on 'Pilonidal cyst || id:finngen_R12_L12_PILONIDALCYST'
